# Mouse Zygotes Respond to Severe Sperm DNA Damage by Delaying Paternal DNA Replication and Embryonic Development

**DOI:** 10.1371/journal.pone.0056385

**Published:** 2013-02-19

**Authors:** Joanna E. Gawecka, Joel Marh, Michael Ortega, Yasuhiro Yamauchi, Monika A. Ward, W. Steven Ward

**Affiliations:** Institute for Biogenesis Research, Department of Anatomy, Biochemistry and Physiology, University of Hawaii at Manoa, John A. Burns School of Medicine, Honolulu, Hawaii, United States of America; The Babraham Institute, United Kingdom

## Abstract

Mouse zygotes do not activate apoptosis in response to DNA damage. We previously reported a unique form of inducible sperm DNA damage termed sperm chromatin fragmentation (SCF). SCF mirrors some aspects of somatic cell apoptosis in that the DNA degradation is mediated by reversible double strand breaks caused by topoisomerase 2B (TOP2B) followed by irreversible DNA degradation by a nuclease(s). Here, we created zygotes using spermatozoa induced to undergo SCF (SCF zygotes) and tested how they responded to moderate and severe paternal DNA damage during the first cell cycle. We found that the TUNEL assay was not sensitive enough to identify the breaks caused by SCF in zygotes in either case. However, paternal pronuclei in both groups stained positively for γH2AX, a marker for DNA damage, at 5 hrs after fertilization, just before DNA synthesis, while the maternal pronuclei were negative. We also found that both pronuclei in SCF zygotes with moderate DNA damage replicated normally, but paternal pronuclei in the SCF zygotes with severe DNA damage delayed the initiation of DNA replication by up to 12 hrs even though the maternal pronuclei had no discernable delay. Chromosomal analysis of both groups confirmed that the paternal DNA was degraded after S-phase while the maternal pronuclei formed normal chromosomes. The DNA replication delay caused a marked retardation in progression to the 2-cell stage, and a large portion of the embryos arrested at the G2/M border, suggesting that this is an important checkpoint in zygotic development. Those embryos that progressed through the G2/M border died at later stages and none developed to the blastocyst stage. Our data demonstrate that the zygote responds to sperm DNA damage through a non-apoptotic mechanism that acts by slowing paternal DNA replication and ultimately leads to arrest in embryonic development.

## Introduction

It is not yet clear how the mammalian zygote responds to DNA damage. Studies on zygotes with induced DNA damage have demonstrated that they do not have traditional G1/S or G2/M checkpoints [1], [2], suggesting that alternative mechanisms are in place to ensure the integrity of the genome in developing embryos. Both non-homologous end joining (NHEJ) and homologous recombination (HR) repair pathways are active in the zygotes and some DNA repair is possible [3]–[5]. Apoptosis, the common response to unrepairable DNA damage in somatic cells, does not appear to be active in mammalian zygotes. It does play a role in eliminating defective embryos, although not until later in embryonic development. Some aspects of apoptosis, such as cytoplasmic fragmentation, occur as early as in the first cell cycle in mice and the second cell cycle in humans [6]–[8]. However, other typical characteristics of apoptosis, including chromatin and cytoplasm condensation followed by DNA degradation and cell shrinkage as well as marginalization and nuclear fragmentation [6], have not been observed until the morula and blastocyst stages [8]–[12]. One of the most commonly used hallmarks for apoptosis, DNA degradation measured by terminal deoxynucleotidyl transferase dUTP nick end labeling (TUNEL), was not detected in bovine embryos before the 6- to 8-cell stage [9], [12]. Fear *et al.* [13], recently reported the developmental changes in the expression of six BCL2 family proteins involved in regulation of apoptosis. They concluded that anti-apoptotic protection exists in the early embryo (2- to 8-cell stages) and is then followed by the establishment of apoptotic capacity at later stages of embryonic development.

How, then, does the embryo respond to DNA damage? It is clear (see below) that embryos cannot survive with severe DNA damage, and in extensive cases these embryos do not progress to the stages in which apoptosis can be activated. In at least one aspect, mouse zygotes appear to be more sensitive to DNA damage than most cells. The histone variant H2AX is present in most cell types as a low percentage, roughly 2%, of the total H2A, and is phosphorylated at serine 139 in response to DNA damage (the phosphorylated form is termed γH2AX) [14], [15]. In mouse zygotes, however, H2AX is the predominant form of the histone H2A [16], raising the possibility that mouse embryos are unusually sensitive to DNA damage. By this measure, zygotes have the capacity to recognize damaged DNA and respond to its presence, but this potential response is complicated by the fact that after fertilization the sperm and oocyte DNA are sequestered into two different pronuclei [17]. DNA replication proceeds in each pronucleus separately before the parental genomes fuse at mitosis. Barton et al. [18] found that when cyclophosphamide treated spermatozoa were used to fertilize normal untreated oocytes only the paternal pronuclei exhibited γH2AX staining [18]. When mouse spermatozoa were irradiated prior to fertilization, a similar pattern was observed, although the intensity of γH2AX signal in the paternal pronuclei appeared to be much lower [19]. However, studies that used gamma [20] or UV irradiation [21] to induce zygote DNA damage found that H2AX phosphorylation occurred only later in development. We have previously shown that injection of spermatozoa with severe DNA damage leads to developmental arrest at stages before apoptosis can be activated [22], [23]. These data suggest that various types of DNA damage elicit different responses from the zygote, some eliciting apoptosis during later stages of embryonic development and others arresting development through non-apoptotic mechanisms. Understanding how the embryo responds to different levels and types of DNA damage, particularly those that lead to early embryonic developmental arrest in the absence of apoptosis will provide important insight into the early development of the embryo, and may lead to the identification of novel cell cycle arrest or cell death mechanisms.

The ability to introduce DNA damage in the spermatozoon before it is allowed to fertilize provides a unique system for studying the response of the oocyte to genetic aberrations. Of particular interest is whether DNA damage in the paternal pronucleus has any effect on the maternal pronucleus. In the study by Barton et al. [18], only the paternal pronuclei phoshorylated H2AX, but both paternal and maternal pronuclei had increases in poly(ADP-ribose) polymerase-1 (PARP-1), a protein involved in later stage of DNA damage response, suggesting that some cross-talk exists between the two pronuclei [18]. Another example of pronuclear cross-talk was described by Shimura et al., who used irradiated mouse sperm to fertilize normal untreated oocytes and found that both pronuclei exhibited p53 DNA damage responses and replicated only about half of their DNA [2], [24]. This finding was remarkable in that the embryos progressed through later stages of development, dying only after implantation. In somatic cells, the p53 dependent S-phase checkpoint leads to cell cycle arrest and then apoptosis if the damaged DNA cannot be repaired [25]. The authors concluded that the p53 dependent S-phase checkpoint in the zygotes marked the DNA for delayed induction of apoptosis.

**Figure 1:**
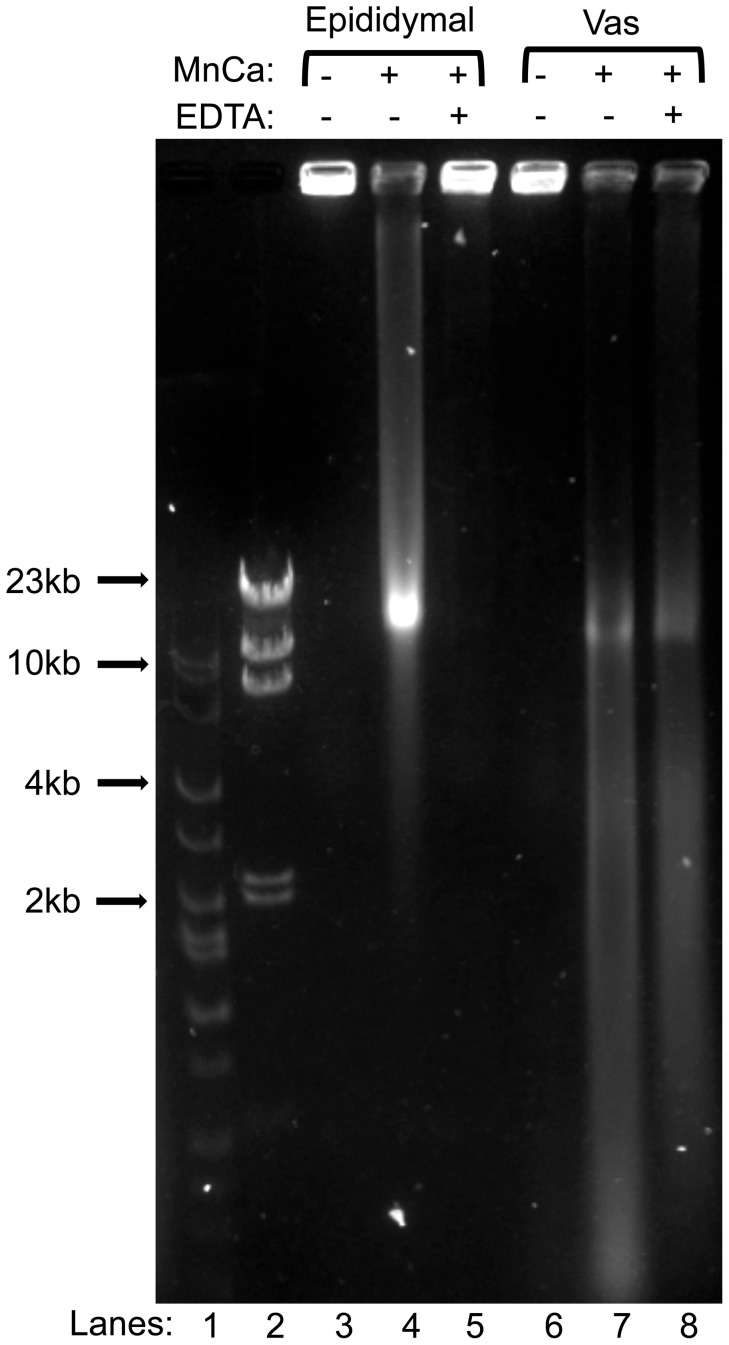
DNA degradation by divalent cations in vas deferens sperm is more severe and less reversible than in epididymal sperm. Spermatozoa from the epididymides (lanes 3–5) or the vas deferens (lanes 6–8) were embedded in agarose plugs, and incubated in mHCZB supplemented with MnCl_2_ and CaCl_2_ as described in Methods, for 1.5 hrs at RT. They were then treated without EDTA (lanes 4 and 7) or with (lanes 5 and 8) to reverse TOP2B-induced breaks. The plugs were then electrophoresed by FIGE. Control lanes (3 and 6) were untreated spermatozoa. Lines 1 and 2 are markers.

We have previously reported that when mouse sperm are treated with divalent cations they degrade their DNA in an apoptotic-like manner, described as sperm chromatin fragmentation (SCF) [26]. We have also shown that spermatozoa from vas deferens degraded their DNA to a greater extent than spermatozoa from caudae epididymides [23] (see also Figure 1), providing a model for two different degrees of sperm DNA damage that can be induced within the sperm cell. In this study, we injected spermatozoa that had been induced to undergo SCF (SCF-spermatozoa) into oocytes and followed the zygotes through the first cell cycle, and the resulting embryos through early development. We demonstrated that zygotes recognize and respond to sperm DNA damage through a unique non-apoptotic mechanism featuring delay in DNA replication, retarded embryonic development and ultimately developmental arrest.

## Materials and Methods

### Antibodies

The following kits or antibodies were used: TUNEL In Situ Cell Death Detection Kit, POD (Roche #11-684-817-910), γH2AX S139 (Millipore #05-636), DNA Replication Click-iT EdU HCS Assays (Invitrogen # C10350).

### Animals

B6D2F1 (C57BL/6N × DBA/2) mice were obtained from the National Cancer Institute (Raleigh, NC). Mice were kept in accordance with the guidelines of the Laboratory Animal Services at the University of Hawaii and those prepared by the Committee on Care and Use of Laboratory Animals of the Institute of Laboratory Resources National Research Council [27]. The protocols for animal handling and the treatment procedures were reviewed and approved by the Institutional Animal Care and Use Committee at the University of Hawaii.

### Preparation of Spermatozoa for Intracytoplasmic Sperm Injection (ICSI) and Field Inversion Gel Electrophoresis (FIGE)

Spermatozoa were collected and treated as previously described [28]. Briefly, spermatozoa were collected in mHCZB (modified Hepes-CZB, CZB media buffered with Hepes and without magnesium, calcium or EDTA) [29] The suspension was mixed by gentle pipetting and divided into groups: control, SCF or SCF-religated. Control spermatozoa were incubated at room temperature (RT) for 1.5 hrs. In the SCF and SCF-religated groups spermatozoa were incubated at RT for 1.5 hrs in the presence of 10 mM MnCl_2_ and 10 mm CaCl_2_. After that, the SCF-religated group was supplemented with 100 mM EDTA and incubated for 30 min at RT. Treated spermatozoa were used for ICSI immediately after the reaction’s time was reached. For FIGE, the suspensions after treatments were mixed by gentle pipetting with low-melting agarose to final concentration of 1% agarose, and poured into molds making ∼5 mm-thick plugs. The plugs were then incubated in the digestion buffer (10 mM Tris, 5 mM EDTA pH 7.8, 100 mM NaCl, 0.5% SDS and 20 mM DTT) for 1.5 hrs at 55**°**C and placed in 0.8% agarose gel for field inversion gel electrophoresis (FIGE).

### Oocyte Collection, ICSI and Embryo Culture

#### Oocyte collection

Mature females, 8–12 wks old, were induced to superovulate with intraperitoneal injections of 5 IU eCG and 5 IU hCG given 48 hrs apart. Oviducts were removed 12–14 hrs after the injection of hCG and placed in HCZB medium [30], [31]. The cumulus-oocyte complexes were released from the oviducts into 0.1% of bovine testicular hyaluronidase (300 USP units/mg) in HCZB to disperse cumulus cells. The cumulus-free oocytes were washed with HCZB medium and used immediately for ICSI.

#### Intracytoplasmic sperm injection (ICSI)

ICSI was carried out as described by Szczygiel and Yanagimachi [30], [32]. A small drop of treated sperm suspension was mixed thoroughly with an equal volume of HCZB containing 12% (w/v) polyvinyl pyrolidone (PVP, M_r_ 360kDa) immediately before ICSI; a single sperm head was injected into each oocyte. ICSI was performed using Eppendorf Micromanipulators (Micromanipulator TransferMan, Eppendorf, Germany) with a Piezo-electric actuator (PMM Controller, model PMAS-CT150, Prime Tech, Tsukuba, Japan).

#### Embryo culture

After ICSI, oocytes were cultured in CZB [33] for the indicated time at 37°C and in 5% CO_2_ in air.

### Immunocytochemistry

#### Assessment of DNA replication

DNA replication analysis was performed by assaying the incorporation of 5-ethynyl-2′-deoxyuridine (EdU) according to the manufacturer’s protocol (Invitrogen, Click-iT EdU HCS assay kit) with modifications. Briefly, immediately after ICSI oocytes were transferred to fresh media containing 1X EdU component A (Invitrogen, Click-iT EdU HCS assay kit) for an additional time, as indicated. EdU component A was prepared as a 100X stock solution in CZB, and 1 µl was added to 99 µl of media for EdU incubation. Following incubation in EdU, the oocytes were rinsed in PBS and fixed with 4% paraformaldehyde in PBS for 30 mins at RT. Fixed oocytes were washed 3 times in PBS and stored in PBS under mineral oil for further analysis. The oocytes were then permeabilized in 0.1% TX in PBS for 15 mins at RT, washed 3×5 mins in PBS and incubated for 30 mins at RT in Click-iT reaction cocktail (prepared following the manufacturer’s protocol). After the incubation, the oocytes were washed once for 5 mins with Click-iT rinse buffer (component F), followed by two 5 mins washes with PBS. The oocytes were placed on the microscope slides and allowed to dry. The preparations were covered with Prolong Gold Antifade mounting media containing DAPI (Invitrogen) and examined using a fluorescence microscope fitted with the appropriate filters. Maternal and paternal pronuclei were differentiated by the fact that the paternal pronucleus in the mouse is larger [34].

#### Assessment of DNA damage by TUNEL

At indicated time after ICSI the oocytes were rinsed in PBST (0.1% Tween in PBS) and fixed for 30 mins at RT in 4% paraformaldehyde. The oocytes were then washed three times for 5 mins with PBST, and stored in PBST under mineral oil for further analysis. The oocytes were then permeabilized for 1 h in freshly prepared 0.1% TX in 0.1% sodium citrate at RT. The oocytes were then transferred to TUNEL detection reagent (In Situ Cell Death Detection Kit, Roche) and incubated under mineral oil 1 h or overnight at 37**°**C (no differences in staining pattern were noted). After the labeling the oocytes were washed three times for 10 mins with PBST, transferred to microscopy slides, allowed to dry and mounted with Prolong Gold Antifade mounting media containing DAPI (Invitrogen) as described above. For positive control, fixed oocytes were incubated with 20 U DNAse I in CZB supplemented with 0.5 mM CaCl_2_ for 1 h at 37**°**C, followed by thorough washing with PBS, permeabilization and further labeling.

#### γH2AX immunostaining

At indicated time after ICSI the oocytes were fixed as described above for the TUNEL assay. The oocytes were then permeabilized in PBST, and washed twice in PBST+0.5% BSA for 10 mins each wash. The oocytes were then blocked in 5% BSA/PBS for 1 h, followed by primary antibody labeling performed overnight at 4**°**C. Three 10 mins washes in PBST+0.5% BSA were then followed by secondary antibody labeling performed for 1–2 hrs at RT. The oocytes were then rinsed in PBST, washed twice in PBST+0.5% BSA for 10 mins each wash. After the labeling the oocytes were washed three times for 10 mins with PBST, transferred to microscopy slides, allowed to dry and mounted with Prolong Gold Antifade mounting media containing DAPI (Invitrogen) as described above.

#### Analysis of EdU staining

The staining intensity for EdU incorporation in the paternal pronuclei was compared to the maternal pronuclei in the same zygotes. EdU immunofluorescence was analyzed using NIH image analysis software Image J (available at http://rsbweb.nih.gov/ij/). The brightness of gray scale-converted EdU signal in a vacuole-free region of interest (ROI) in paternal pronucleus was compared to the same sized, vacuole free ROI in the maternal pronucleus. For Vas-Ctrl zygotes, the ratio of the EdU signal in the paternal pronucleus to that of the maternal pronucleus was 0.975±0.22 (MEAN ± SD). For this analysis, we conservatively defined zygotes in which the paternal pronucleus and the maternal pronucleus had similar EdU signal intensities as zygotes that had a paternal to maternal EdU signal ratio of 0.5 or higher.

### Chromosome Analysis

Chromosome analysis was performed as previously described [35]. Briefly, fertilized oocytes were transferred after 6 to 8 hrs of culture into CZB containing vinblastine at 0.006 µg/ml to inhibit syngamy. Between 19 and 21 hrs after ICSI, oocytes were treated with 1% pronase (1000 tyrosine units/mg; Kaken Pharmaceuticals, Tokyo, Japan) for 5 mins at RT to soften the zona pellucida. The oocytes were then treated with hypotonic solution (1∶1 mixture of 1% sodium citrate and 30% fetal bovine serum) for 5 mins at 37°C or 10 mins at 25°C. Chromosomes were spread on clean glass slides by the gradual fixation/air-drying method [36]. The preparations were stained with 2% Giemsa (Merck, Darmstadt, Germany) in PBS (pH 6.8) for 10 min for conventional chromosome analysis. The chromosomes of a spermatozoon were considered normal when an oocyte contained 40 normal metaphase chromosomes. It was not always possible to distinguish between chromosomes of paternal and maternal origin. However, because oocyte chromosomes rarely show structural aberrations at first cleavage metaphase after parthenogenetic activation [22], any abnormal chromosomes within fertilized oocytes were believed to be of sperm origin.

### Statistical Analysis

In the examination of embryonic damage (γH2AX immunostaining) and DNA replication (EdU immunostaining) the percentage of stained embryos in all subgroups was analyzed and compared. Experiments within each treatment were repeated two to three times. The variation between experiments within the same treatment was determined by calculating the percentage of stained embryos in each experiment, taking the mean, and calculating the SD. For comparing the different treatment groups, the Student’s T test and/or 1 way ANOVA analysis was performed. Significance was determined at *p<0.05, **p<0.01, ***p<0.001. For chromosomal analysis, the Fisher's exact test was used for analyzing the differences between groups.

## Results

### Cauda Epididymal and vas Deferens Spermatozoa Fragment their Chromatin to Different Extents in Response to Mn^2+^/Ca^2+^ Treatment

We have previously reported that mature spermatozoa have the ability to digest their own DNA into loop-size fragments upon incubation with Mn^2+^/Ca^2+^ [23], [28], [37], [38] and that this ability is reversible by the presence of EDTA [26]. We observed that spermatozoa from vas deferens had more extensive sperm chromatin fragmentation (SCF) than spermatozoa from cauda epididymides [28]. Furthermore, in vas deferens spermatozoa the reversibility of this fragmentation was minimal, while in epididymal spermatozoa it was nearly complete [26]. This suggests that the nuclease in vas deferens sperm is much more active than in epididymal spermatozoa. Here, we confirm these previously reported differences in SCF activity and reversibility between vas and epididymal spermatozoa under the modified conditions (see Materials and Methods) that were used throughout the experiments described in this work (Fig. 1).

### DNA Fragmentation in SCF Spermatozoa is not Detectable by TUNEL after Fertilization

**Figure 2:**
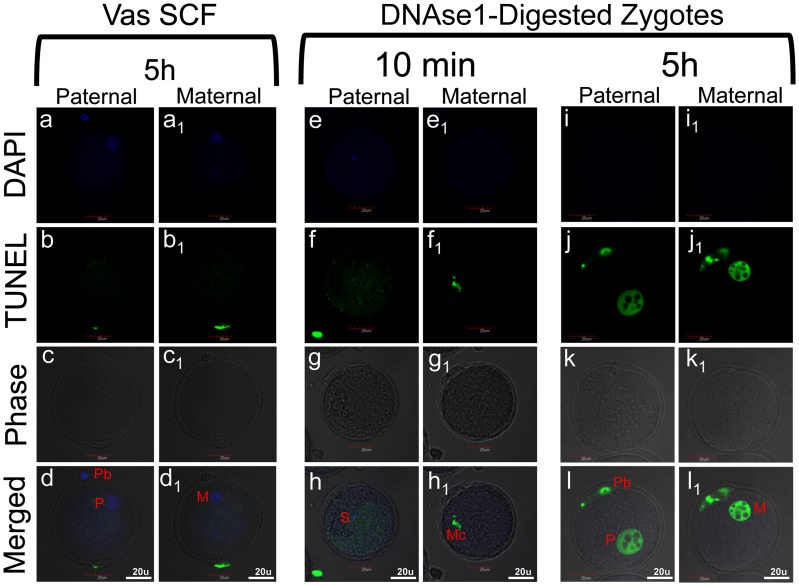
DNA fragmentation induced in SCF spermatozoa is not detectable by TUNEL after fertilization. (a–d_1_) A Vas-SCF zygote stained with TUNEL and counterstained with DAPI 5 hrs after sperm injection, and visualized by confocal microscopy. (e–h_1_) A control zygote that was fixed and treated with DNAse I 10 mins after fertilization, and then stained by TUNEL. (i–l_1_) A control zygote that was treated with DNAse I 5 hrs after fertilization. Double columns in each panel present the same embryo at different focal planes. S – sperm, Mc – maternal chromatin, M – maternal pronucleus, P – paternal pronucleus, Pb – polar body. All images are shown at the same magnification (bars = 20 µm).

As described in the Introduction, it is currently thought that in early embryos apoptosis is actively inhibited, followed by the establishment of apoptotic capacity at later stages of embryonic development [13]. We tested whether injecting oocytes with sperm bearing known DNA damage (which prevents the embryos from further survival as previously reported [23]) results in paternal pronuclei that are sensitive to the TUNEL reaction. SCF-spermatozoa are not motile and cannot fertilize oocytes on their own, so fertilization can only be achieved by ICSI. The sperm that were injected had double stranded DNA breaks resulting in the entire genome being fragmented to sizes of 25 kb and smaller, with some larger fragments still visible (Fig. 1). We examined control zygotes created by injecting oocytes with control epididymal sperm at 1 hr (n = 8), 5 hrs (n = 17), 9 hrs (n = 20), and 20 hrs (n = 13) after fertilization, and Vas-SCF zygotes, the zygotes with the highest degree of DNA damage, (n = 11, 11, 17 and 7, respectively for the same time points). We found no signs of TUNEL labeling in any of these embryos. One example, a Vas-SCF zygote labeled at 5 hrs post-injection, is shown in Fig. 2a–d_1_. This suggests that either the level of DNA damage in the paternal pronuclei was below the level of detection for TUNEL or the damage was repaired in the oocyte. We consider the latter scenario unlikely for the reasons discussed below.

To test our hypothesis that the DNA damage in SCF sperm was below the level of detection by TUNEL we treated normal zygotes with DNAse I at various time points after permeabilization but before fixation for TUNEL. When rodent spermatozoa are treated with DNAse I, the chromatin is fragmented to 25–50 kb because most of the sperm chromatin is protected from nuclease digestion by protamines [37]. Therefore, the DNAse I-induced breaks in the sperm would be expected to be on the same order of magnitude as those in SCF-induced sperm. We have shown earlier that 30 mins after injection the majority of sperm (∼70%) retain unchanged chromatin which has not yet started to decondense [39]. Thus, with respect to the chromatin status, normal, untreated spermatozoa before injection and 10 mins after injection are nearly identical. When we treated zygotes, obtained after ICSI with such spermatozoa, with DNAse I at 10 mins post-ICSI sperm heads did not stain positively with TUNEL (Figure 2e–h). In the same zygote, the maternal chromosomes, which are histone bound and therefore much more susceptible to DNAse I digestion, served as a positive internal control for the TUNEL assay. DNAse I digests histone bound chromatin into fragments far smaller than 25 kb. The maternal chromatin was more extensively degraded by DNAse I and was detectable with TUNEL (Fig. 2f_1_). At 5 hrs after injection sperm heads have already transformed into pronuclei and sperm chromatin have undergone protamine-to-histone exchange. This remodeled paternal chromatin was now similarly sensitive DNAse I treatment as maternal chromatin (Fig. 2j–j_1_). One possible explanation for these results is that the TUNEL assay cannot detect DNA damage when the chromatin is fragmented to 25 kb but it does when the degradation is more extensive.

### DNA Fragmentation in SCF Spermatozoa is Recognized by the Zygote

**Figure 3:**
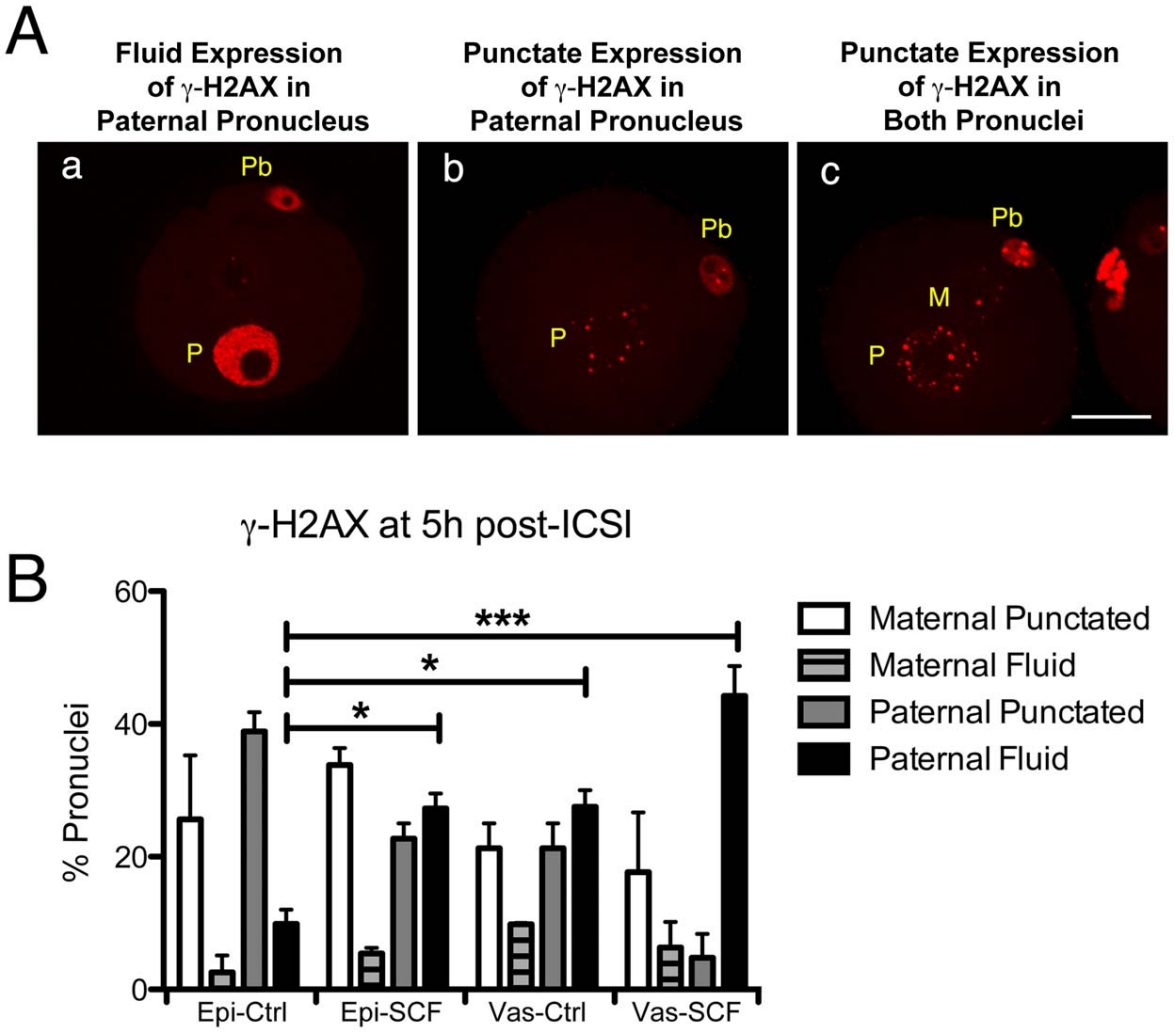
γH2AX is detectable in the paternal pronuclei of Vas-SCF zygotes before DNA synthesis. (A) Vas-SCF zygotes showing three different patterns of γH2AX staining. (Aa) Fluid expression of γH2AX in the paternal pronucleus (P) and no visible expression in the maternal pronucleus; (Ab), Punctate γH2AX expression in the paternal pronucleus; (Ac), Punctate expression in both pronuclei. (B) Four groups of embryos were analyzed and scored for γH2AX staining in each pronuclei (n = 30 to 47 zygotes in each group).

We next tested whether the zygote exhibited any evidence of recognizing DNA damage in sperm DNA by testing for H2AX phosphorylation. We noticed two distinct patterns of γH2AX. One type was intense, even staining throughout the pronucleus with the exception of the nucleoli, that we term the fluid pattern (Fig. 3Aa). This is the type of γH2AX staining that is typically associated with DNA damage [18], [40]. The second was very low levels of punctate staining dispersed in the pronucleus which appeared either in paternal (Fig. 3Ab) or both paternal and maternal (Fig. 3Ac) pronuclei. This type of staining has not been widely reported and is not necessarily associated with DNA damage [41]. We found that in zygotes created by injecting untreated epididymal spermatozoa (Epi-Ctrl zygotes), both pronuclei often had low levels of punctate γH2AX staining while far fewer had the intense, fluid staining throughout the pronucleus (Fig. 3B). Even in these normal embryos, however, the paternal pronucleus was 3.85 times more likely to have the fluid pattern of staining (9.86% versus 2.56%). In Epi-SCF zygotes, the percentage of embryos with fluid γH2AX staining rose 2.8 fold, but did not change significantly in the maternal pronuclei. A high percentage of zygotes created by injecting normal vas deferens spermatozoa (Vas-Ctrl zygotes) also had paternal pronuclei with fluid γH2AX staining, and this almost doubled in Vas-SCF embryos. These data suggest that DNA damage in SCF-spermatozoa persists, and it is detectable in the zygotes with γH2AX staining. The data also support an earlier report showing that mouse embryos are extremely sensitive to H2AX phosphorylation even in the absence of induced DNA damage [42].

### DNA Replication is Delayed in Paternal Pronuclei with Fragmented DNA

**Figure 4:**
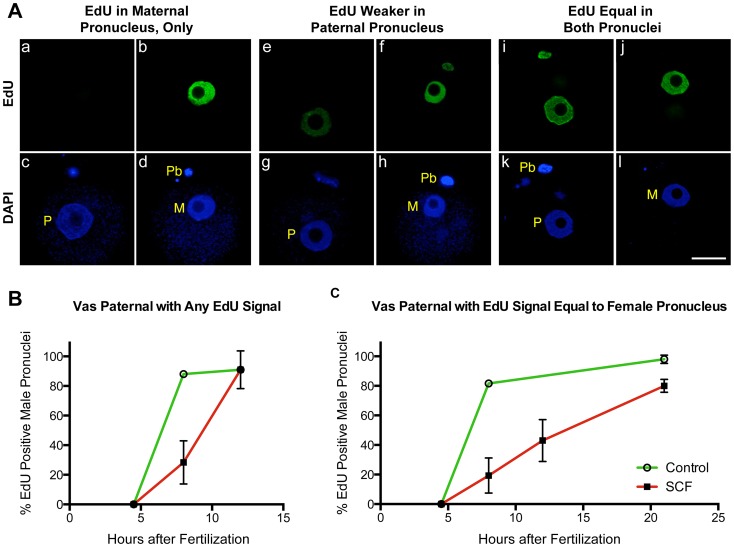
DNA replication is delayed in Vas-SCF zygotes. (A) Three different patterns of EdU staining in Vas-SCF zygotes during S-phase. Each set of four images represents the same zygote at different focal planes to view the paternal (right) and maternal (left) pronuclei. (Aa-d) In most cases during early S-phase, EdU staining was only visible in the maternal pronucleus. (Ae-h) EdU staining was much more intense in the maternal pronucleus, but was it was also visible in the paternal. (Ai-l) Edu staining was visible at equal intensity in both pronuclei. All images are shown at the same magnification, bar = 20 µm. (B) Zygotes with any EdU staining in the paternal pronucleus, whether it was much less or equal to that of the maternal pronucleus, were scored as positive. (C) Only zygotes that had paternal pronuclei with EdU staining intensities equal to that of the maternal pronuclei in the same zygote were scored only as positive. Error bars represent standard deviations, and points on the graphs for which the error bars are not visible had standard deviation values below the size that is covered by the point drawn to indicate the value. For at 50 Vas-SCF zygotes were scored for each time point, and 30 Vas-Ctrl zygotes were scored. In all zygotes at 8 hrs and beyond, maternal pronuclei had strong EdU staining. All images are shown at the same magnification, bar = 20 µm.

We next tested an alternate explanation for our previous finding that paternal pronuclei in SCF zygotes do not replicate their DNA. It was possible that they did initiate DNA replication, but with a long delay as compared to the maternal pronuclei. Normally, both pronuclei initiate DNA synthesis relatively synchronously between 6 to 7 hrs after fertilization [39], [43]. We first confirmed that Epi-SCF zygotes replicated normally, as we had previously reported [23]. Epi-SCF zygotes were incubated with EdU, and all zygotes had equal intensity EdU staining in both pronuclei by 8 hrs after fertilization. We next incubated Vas-SCF zygotes with EdU at different time points for up to 21 hrs after sperm injection. We noticed three distinct patterns of EdU staining in the paternal pronuclei. During the first few hours of DNA synthesis, the EdU signal was often absent in the paternal pronucleus when maternal pronucleus had strong EdU staining (Fig. 4Aa–d). By 12 hrs after fertilization, 46% of the Vas-SCF zygotes had paternal pronuclei clearly positive but with a much lower intensity of EdU staining than the maternal pronuclei in the same embryo (Fig. 4Ae–h). For the purposes of this analysis, these were conservatively defined as zygotes with a paternal pronucleus to maternal pronucleus EdU staining intensity ratio of <0.5 as determined by image analysis (see Methods). At this same time point, both pronuclei exhibited equal EdU staining intensity in 43% of the zygotes (defined as zygotes with an EdU staining intensity ratio of ≥0.5) (Fig. 4Ai–l).

These data are shown graphically in Fig. 4B and C. To avoid any bias resulting from choosing a zygote with lower paternal EdU staining intensity, we first plotted the percentage of Vas-SCF and embryos created by injecting normal vas deferens spermatozoa (Vas-Ctrl zygotes) that had any positive signal in the paternal pronuclei (Fig. 4B). As previously described [23], we did not observe any replication at 4.5 hrs post-injection in either SCF or control zygotes. We also confirmed our previous observation that the majority of paternal pronuclei in Vas-SCF zygotes failed to replicate by 8 hrs post-injection (Figure 4B) while all maternal pronuclei were strongly positive for EdU staining. However, when the zygotes were followed for longer times, most of the paternal pronuclei did initiate DNA repliction, albeit much delayed. There was a lag of up to 4 hrs in the initiation of DNA replication in the paternal pronuclei in the SCF group as compared to the control. We next plotted the percentage of zygotes that had equal EdU staining in both pronuclei over time (Fig. 4C). This experiment was conducted by incubating the embryos continuously in EdU, so the intensity represents the total DNA synthesis up to that time point. In this case we found that there was an almost linear increase in the percentage of Vas-SCF zygotes that contained two pronuclei with similar EdU intensities. Overall, these data indicate that paternal pronuclei with severely damaged DNA initiate DNA replication but several hours after the maternal pronuclei.

### SCF Sperm DNA Damage Persists after DNA Replication

**Figure 5:**
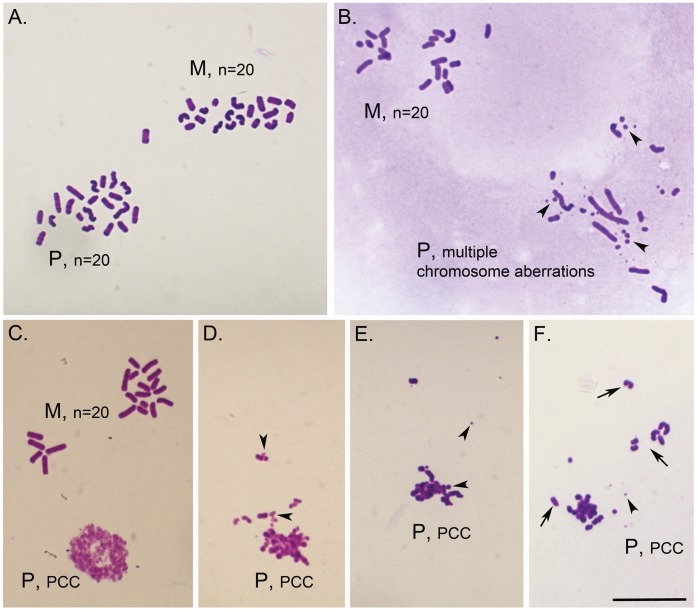
Chromosomal analysis of Vas-SCF and Epi-SCF zygotes. (A) Vas-Ctrl; normal maternal (M) and paternal (P) chromosome complements, n = 20 chromosomes each. Maternal and paternal chromosome plates can be distinguished based on chromosome morphology; maternal chromosomes are usually shorter and thicker. (B) Epi-SCF; normal maternal chromosome complement (M, n = 20) and paternal complement (P) with multiple chromosome aberrations. Some of the chromosome fragments are shown with arrowheads. (C) Vas-SCF, normal maternal complement (M, n = 20 chromosomes) and paternal chromatin seen as early stage of prematurely condensed chromosomes (PCC). (D-F) Vas-SCF; examples of paternal chromatin at different levels of premature chromosome condensation (PCC). Note the increasing chromatin condensation and formation of few separate chromosomes (arrows), from D to F, as well as presence of chromosome fragments (arrowheads). Scale = 20 µm.

The fact that paternal pronuclei in Vas-SCF zygotes did replicate DNA with a significant delay raised the possibility that the embryos were able to repair the fragmented sperm DNA. Therefore we examined the chromosomes after DNA replication to test for persistent paternal DNA damage. We found that in zygotes created with normal, epididymal spermatozoa all of the maternal chromosomes and most (87%) of the paternal chromosomes were normal (Table 1, Fig. 5A). When normal vas deferens sperm were used for injection, the number of oocytes with normal paternal chromosomes was slightly reduced (69%), consistent with our previous findings that vas deferens sperm were less efficient in ICSI [23], although this was not statistically significant. In Epi-SCF zygotes, only 24% of the paternal karyoplates were normal, even though all the maternal karyoplates were (p<0.01). Most of these aberrations were chromosome breaks, with an aberration rate, reflecting the severity of chromosome damage, of 5.82 per karyoplate (see Fig. 5B for an example). In Vas-SCF zygotes, all of the maternal chromosomes were normal, but the large majority (77%) of the paternal karyoplates had prematurely condensed chromatin (PCC, see Fig. 5C-F for examples of PCC in SCF zygotes) [44], [45]. The few zygotes that did have analyzable chromosomes, had them largely fragmented (Table 1). Out of 66 Vas-SCF zygotes examined, only one had normal paternal chromosomes.

**Table 1:** Paternal Chromosomal analysis of zygotes obtained after ICSI with SCF-induced sperm.

SpermSampleInjected	No of Zygotes Analyzed^a^	With PCC No (%)^b^	With Analyzable ChromosomesNo (%)	NormalNo (%)^c^	AbnormalNo (%)^c^	Aberration Rate^d^
Epi-Ctrl	31	1 (3.2)	30 (96.8)	26 (86.7)	4 (13.3)	0.37
Epi-SCF	39	1 (2.6)	38 (97.4)	9 (23.7)**	29 (76.3)	5.82
Vas-Ctrl	27	1 (3.7)	26 (96.3)	18 (69.2)	8 (0.8)	0.46
Vas-SCF	66	51 (77.3)*	15 (22.7)	1 (6.7)**	14 (93.3)	6.80

All zygotes analyzed had normal maternal chromosomes.

a Data shown represent pooled data from two to four replicates performed for each experimental group.

b Percent calculated from number of zygotes analyzed.

c Percent calculated from number of analyzable paternal chromosomes.

d Aberration rate represents the total number of aberrations divided by the number of oocytes examined and is an indicative of the severity of chromosome damage.

Statistical significance (Fisher's Exact Test, P<0.001):

* different than all others within column;

** different than controls.

### Embryos Generated with SCF Sperm Exhibit Developmental Delay and Arrest Paralleling the Level of DNA Damage

During the chromosomal analysis we noticed another problem with the Vas-SCF zygotes. In those experiments, out of 244 oocytes that were fertilized with ICSI with SCF-induced sperm from vas deferens, 151 (62%) were still in the zygotic two pronuclear stage (2PN) at 19–21 hrs after fertilization when most normal embryos already progress to mitosis. In Vas-Ctrl zygotes only 5.8% (4/69) zygotes were still at the 2PN stage. This suggested that either the DNA damage in vas deferens SCF-spermatozoa was severe enough to inhibit the majority of zygotes from progressing beyond the 1-cell stage, or that the development of Vas-SCF zygotes was markedly delayed. We tested this by following the development of Epi-SCF and Vas-SCF zygotes as compared to Epi-Ctrl and Vas-Ctrl zygotes. In particular, we wanted to identify the step of early development at which Vas-SCF zygotes either arrested or progressed more slowly.

**Figure 6:**
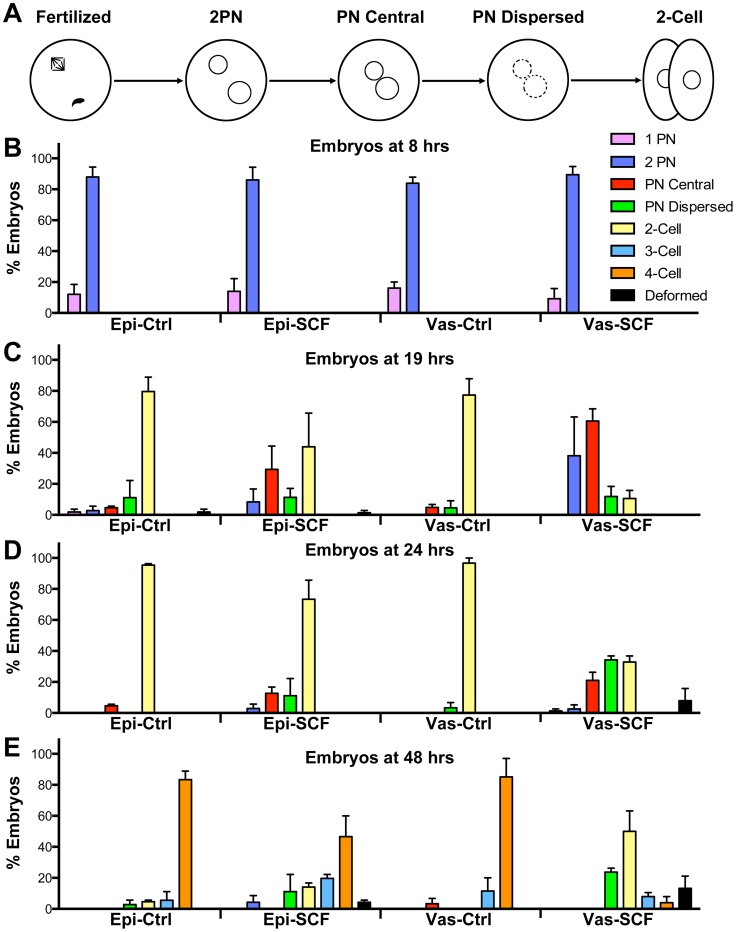
Developmental arrest in embryos with damaged DNA. Embryos produced by injection of Epi-Ctrl, Vas-Ctrl, Epi-SCF and VAS-SCF were closely followed for four days in culture, and scored for their stage of development. (A) Diagram of the early (first cycle) stages of development that were scored. Quantitative data showing the developmental progress at 8 (B), 19 (C), 24 (D), and 48 (E) hrs after fertilization. Fertilization was achieved by ICSI with injections timed not to exceed 15 min per group. For explanation of developmental stages see text. The experiment was repeated twice with at least 45 embryos examined for each group. Error bars represent standard deviations.

We divided zygotic development into four stages (Fig. 6A). Shortly after fertilization, the maternal chromosomes of the second meiotic metaphase separate, and one half form the maternal pronucleus. The sperm nucleus decondenses to form the paternal pronucleus. These events are not synchronized exactly, so that in scoring embryos there are occasionally zygotes with only one pronucleus (1PN). The presence of one pronucleus only, which almost certainly is maternal, may also indicate that sperm succeeded in activating the oocyte but failed undergoing chromatin remodeling and forming a paternal pronuclei. By 4 hrs after fertilization, both pronuclei are formed, and are usually separated in the cytoplasm (2PN stage). By 12 to 15 hrs after fertilization, the two pronuclei have migrated to a central position in the zygote (PN central stage). Shortly before cleavage, the two pronuclear membranes are disrupted as the chromosomes start to condense, and the pronuclei are no longer visible (PN dispersed stage). The zygote then proceeds through cleavage, and onto the 2-cell stage.

We found that zygotes responded to severe paternal DNA damage by slowing progression during the first divisions. At 8 hrs after fertilization, most of the embryos had developed to the 2PN stage with no discernible difference between the groups (Fig. 6B). By 19 hrs after injection differences between the groups started to appear. The large majority of Epi-Ctrl and Vas-Ctrl embryos had progressed to the 2-cell stage (Fig. 6C). Most of the Epi-SCF embryos had also progressed beyond the 1-cell stage, but 29.4% remained at the PN central stage. At the same time, the majority of the Vas-SCF embryos were still at the 1-cell stage (60.5% at the PN central stage and 22.4% at the PN dispersed stage). A similar trend was seen at 24 hrs post injection (Fig. 6D). By this time point, most of the embryos in both control, and Epi-SCF groups had progressed to the 2-cell stage, but only 32.9% of the Vas-SCF embryos cleaved, with the majority (59.2%) remaining at the 1-cell stage. By 48 hrs after sperm injection, when most of the control embryos and the majority of the Epi-SCF embryos were at the 4-cell stage, most of the Vas-SCF embryos were arrested at the 1 or 2-cell stage (Fig. 6E). At this time point, 23.7% of the Vas-SCF embryos were arrested as 1-cell embryos, all at the PN dispersed stage.

## Discussion

In this study we have shown that mouse zygotes respond to sperm DNA damage through a non-apoptotic pathway. DNA replication in the paternal pronuclei is delayed, as is embryonic development, particularly in the first cell cycle at the G2/M border. Moreover, the level of the response depends on the severity of sperm DNA damage. While the molecular pathways remain to be identified, our data reveal several important aspects of this unique DNA damage response.

### The Zygote Recognizes SCF-induced DNA Damage, but does not Respond by Initiating Apoptosis

SCF-spermatozoa from neither the epididymides nor vas deferens have DNA damage that is recognizable by the TUNEL assay after fertilization (Fig. 2, and text). We tested zygotes as late as five hours after fertilization when the sperm DNA was no longer compacted by protamines [39] but none of the samples were TUNEL positive. It is unlikely that the damaged DNA was fully repaired during the first few hours because our chromosomal analysis demonstrated that chromosome breaks persisted in most cases through mitosis. Thus, the DNA damage in even the most severe cases of SCF was below the level of detection for the TUNEL assay. This is not surprising when one considers that the assay was originally developed to detect apoptotic DNA degradation of the genome to nucleosome-sized fragments of about 0.2 kb [46]. SCF fragments in epididymal spermatozoa are on the order of 25 kb and therefore have far fewer free ends for the TUNEL assay to detect, and those from vas deferens spermatozoa, while smaller than 25 kb, still do not approach the 0.2 kb fragment size. The lack of TUNEL reaction also suggests that this level of DNA damage does not induce further apoptotic DNA degradation that would lead to a positive TUNEL reaction in the zygote apoptosis. This is consistent with reports that mammalian zygotes are not capable of initiating apoptosis [9], [12], [13].

The zygotes did respond to sperm DNA damage by phosphorylating H2AX, roughly in proportion to the severity of DNA damage. As described in the Introduction, H2AX phosphorylation in mammalian zygotes is complicated by the fact that it occurs during normal development without any evidence of DNA damage [42], probably because H2AX is the major H2A variant in these cells [16]. However, we did find clear evidence that in zygotes produced with sperm harboring DNA breaks paternal pronuclei have increased γH2AX levels, as did two other groups using different approaches to induce DNA damage [18], [19]. We found two patterns of γH2AX staining, which we termed 'punctate' and 'fluid', the latter being much more ubiquitous throughout the pronuclei and of higher intensity. McManus et al [41] proposed that low levels of H2AX phosphorylation in normally growing cells were important for maintaining the fidelity of the mitotic process. While the zygotes differ from cells growing in culture in many ways, it is possible that the punctate pattern of γH2AX in normal pronuclei is part of this type of maintenance mechanism, and the greater intensity and more ubiquitous fluid pattern represents a direct response to DNA damage.

### Sperm DNA Damage Results in Replication Delay in the Paternal Pronuclei, but not in the Maternal Pronuclei

Another response of the zygote to severe paternal DNA damage was that in most cases DNA replication was significantly delayed in the paternal pronuclei as compared to the maternal pronuclei. Normally, the paternal and maternal pronuclei in the mouse zygotes initiate DNA replication nearly synchronously between 5 and 6 hrs after fertilization [43], [47]. In the Vas-SCF zygotes, DNA replication was delayed by up to 10 hrs in the paternal, but not the maternal, pronuclei. Our data clearly indicate that the two pronuclei acted independently in arresting DNA synthesis, in response to DNA damage in only one pronucleus. This delay correlates roughly with the level of DNA damage since Vas-SCF zygotes had more extensive paternal DNA damage than Epi-SCF zygotes. Epi-SCF zygotes never exhibited a delay in paternal DNA replication [23]. This may be due to the lower levels of DNA damage than in the SCF-induced vas deferens spermatozoa (Figs. 1 and 5). It is also possible that minor delays in paternal DNA replication did occur in Epi-SCF zygotes that were below the level of detection (the delay in embryonic development, discussed below, supports this possibility). The lack of DNA replication synchrony between the two pronuclei in response to paternal DNA damage results in two pronuclei in the same cytoplasm that are at different stages of DNA replication. In most of the Epi-SCF zygotes, and in all but one of the Vas-SCF zygotes that progressed to mitosis the paternal chromosomes were damaged, suggesting that no significant DNA repair has taken place in the oocytes after fertilization.

This replication delay may be related to the location of SCF damage in the sperm chromatin. SCF occurs at the sites of attachment of the sperm DNA to the nuclear matrix [29]. In somatic cells these sites are also the sites of DNA replication [48]–[50], and we have proposed that the sperm nuclear matrix DNA loop domain organization is inherited by the paternal pronuclei [51]. Other types of sperm DNA damage do not exhibit replication delays. When X-irradiation was used to damage sperm DNA and the spermatozoa used to fertilize oocytes, the resulting zygotes exhibited less total DNA replication in both pronuclei, but no measureable delay [24]. X-irradiation is an external, indiscriminate mediator of nucleic acid breaks while SCF is caused by proteins associated with the spermatozoa and targets specific chromosome sites. Also, we previously damaged sperm DNA by treating extracted sperm nuclei with restriction endonucleases then injecting these into oocytes [52]. The resulting zygotes did not progress, but did replicate DNA at the normal time. These combined data support the hypothesis that the chromatin organization by the sperm nuclear matrix plays an important role in DNA replication in the zygote, and that disruption of these chromosomal sites affects replication.

### Sperm SCF DNA Damage Results in Delayed Development and Arrest at the G2/M Border

Another response to sperm SCF DNA damage was a severe developmental delay and/or arrest of Vas-SCF embryos, and a lesser developmental delay of Epi-SCF embryos, as compared to controls (Fig. 6). Formation of pronuclei was not affected by SCF, but the progression from G2 to metaphase was. The disappearance of the pronuclei prior to cleavage appears to remain coordinated. At 19 hrs after sperm injection, when control embryos were at the 2-cell stage, most of the delayed Vas-SCF zygotes still had two visible pronuclei. We did not observe a late stage zygote with only one pronuclei, which would have indicated one pronucleus entering metaphase before the other. Eventually, all the Vas-SCF zygotes progressed past the PN central stage. However, nearly a quarter of them (23.7%) arrested at the PN disappearance stage and remained at this stage for 72 hrs, suggesting that metaphase is a checkpoint for zygotic progression.

These data suggest that even though there was not a coordinated response between the two pronuclei to the initiation of DNA synthesis, the two pronuclei did act synchronously with respect to the G2/M transition. The maternal pronuclei appeared to delay progression to metaphase after completing DNA synthesis. Then, either the paternal pronuclei completed DNA synthesis and the two sets of chromosomes condensed together, or the maternal pronuclei eventually stimulated the zygote into metaphase before the paternal pronuclei completed replication. Both possibilities are consistent with the high occurrence of PCC in Vas-SCF embryos (77%, Table 1). PCC can be caused by extensive DNA damage [22], but is also characteristic of cells that are forced into mitosis before they complete DNA synthesis [44], [45]. We suggest that both mechanisms play a role in Vas-SCF zygotic developmental delay/arrest, depending on the level of DNA damage in the paternal pronuclei. If the level is very high, the zygote is more likely to proceed to metaphase before the paternal pronuclei has completed its DNA synthesis.

Both possibilities suggest that some coordination, presumably mediated through cytoplasmic signaling, does occur between the paternal and maternal pronuclei in the zygotes. At least three other groups have provided evidence for cross-talk between the two pronuclei in rat [18], [40], [53] and mouse [2], [19] zygotes in the situations when the paternal pronuclei contained some DNA damage, supporting this conclusion. However, the molecular mechanisms that mediate the transition to metaphase in the zygote are not well understood. In Xenopus oocytes, mitosis is activated by Cdc25C dephosphorylating p34cdc2 of the MPF complex [54]. In human cells, Cdc25C is retained in the cytoplasm and translocated to the nucleus to activate mitosis [55]. In this case, there is a clear relationship between molecular events in the cytoplasm and chromatin condensation. In mammalian zygotes, however, it has recently been shown that Cdc25C is retained in the nucleus throughout the cell cycle, but in its inactive, dephosphorylated form before mitosis [56], so it is an unlikely candidate for a possible cytoplasmic signaling link between the two pronuclei.

The embryos that did progress beyond the one cell stage showed evidence of developmental delay or arrest at all later stages in development. The large majority of Vas-SCF embryos that progressed through mitosis arrested at the 2-cell stage and there was clear evidence of delay in Epi-SCF embryos, though they progressed further. At further time points progression was inhibited either by embryo degeneration or developmental arrest, and, as we have previously reported, neither Epi-SCF nor Vas-SCF embryos developed to blastocyst [23].

### A Model for Non-apoptotic Embryonic Arrest in Response to DNA Damage

**Figure 7:**
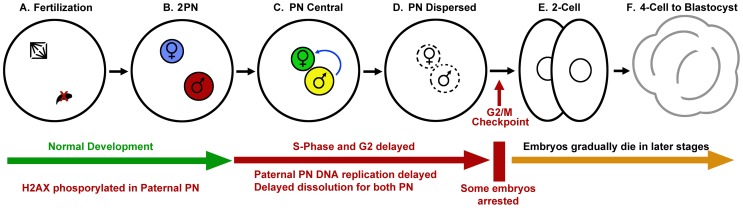
Zygotic response to severe paternal DNA damage. Oocytes fertilized with sperm that have damaged DNA (A) form pronuclei within the normal time, but phosphorylate H2AX in the paternal pronuclei (B). DNA replication is delayed in the paternal but not the maternal pronucleus, and this causes both pronuclei to delay progression to metaphase (C). The pronuclear membranes break down synchronously, though late (D). Some embryos do not progress past the G2/M border, but those that do die or arrest before they progress to blastocysts (F).

Our data, summarized in Fig. 7, indicate that mouse zygotes, even though they do not appear to have the ability to initiate apoptosis, do have a mechanism for modulating embryonic development in response to sperm DNA damage. The formation of the pronuclei proceeds normally, but the paternal pronuclei phosphorylate H2AX indicating that the zygote detects the damage. Paternal DNA replication is delayed by up to 10 hrs even though the maternal pronucleus appears to replicate normally. This causes a coordinated delay of both pronuclei to the G2/M border, indicating communication between the two pronuclei and the cytoplasm. Some of the embryos arrest at this border, suggesting that the G2/M checkpoint can be activated in zygotes. This indicates that sperm DNA damage elicits a specific mechanism to slow early embryonic progression. The embryos that are able to pass through this checkpoint eventually arrest and later die, but at various stages between 2-cell and blastocyst.

We suggest that the architecture of the sperm chromatin plays a central role in this mechanism. The two other studies discussed above using irradiation as a source of sperm DNA damage found no evidence of developmental arrest in mouse embryos [2], [24]. SCF, on the other hand, specifically targets the nuclear matrix attachment regions that are also the putative sites of paternal DNA replication [52]. As discussed above, this would explain the delay in DNA replication in paternal pronuclei. The arrest and death of embryos that pass through the G2/M checkpoint might also be explained by the localization of SCF damage to matrix attachment regions. Hammoud et al. [57] have suggested that genes that are associated with embryonic development, such as HOX genes, FGF9, and SOX7/9, are bound to residual histones in spermatozoa. Histone bound regions in sperm chromatin are the sites that we would expect to be the most susceptible to SCF damage [51], so it is possible that SCF preferentially targets developmental associated genes. This would account for the developmental arrest seen in later stages of development.

Our data suggest that in mice, and potentially other mammals including humans, the structure of the sperm chromatin is part of the mechanism to ensure the transmission of undamaged paternal DNA to the embryo, by interacting with checkpoints in the first cell cycle, particularly at S-phase. This reinforces the necessity to develop strategies for recognition of spermatozoa with abnormal chromatin packaging and/or sperm DNA damage that are frequently observed in infertile men [58], [59] so that such sperm could be avoided in assisted reproduction trials.
